# Bilateral axillary artery cannulation for severely calcified aorta and branches: a case report

**DOI:** 10.1186/s13019-016-0492-1

**Published:** 2016-07-08

**Authors:** Ken Okamoto, Toshihiro Fukui

**Affiliations:** Department of Cardiovascular Surgery, Kumamoto University Hospital, 1-1-1 Honjo, Kumamoto, 860-8556 Japan

**Keywords:** Aortic valve replacement, Aortic calcification, Aortic stenosis, Case report

## Abstract

**Background:**

Aortic valve surgery in patients with severely calcified aortas is technically challenging. Additionally, the choice of arterial cannulation site and whether to perform an aortic clamp to prevent neurological complications are poorly defined.

**Case presentation:**

We describe a patient with a severely calcified aorta and stenosis of its side branches. He successfully underwent aortic valve replacement with bilateral axillary artery cannulation and short-term moderate hypothermic circulatory arrest for cross-clamping of a severely calcified aorta to prevent neurological complications.

**Conclusions:**

Bilateral axillary artery cannulation and short-term moderate hypothermic circulatory arrest for cross-clamping of the porcelain aorta is a suitable option to prevent neurological complications in patients with a severely calcified aorta and stenosis of its side branches who need aortic valve replacement.

## Background

Aortic valve surgery in patients with porcelain aortas is technically challenging. Moreover, no clear guidelines exist regarding the choice of arterial cannulation site and whether to perform an aortic clamp to prevent neurological complications [1]. Herein, we describe a patient with a porcelain aorta and stenosis of its side branches who successfully underwent aortic valve replacement with bilateral axillary artery cannulation and short-term moderate hypothermic circulatory arrest for aortic cross-clamping to prevent neurological complications.

## Case presentation

**Fig. 1:**
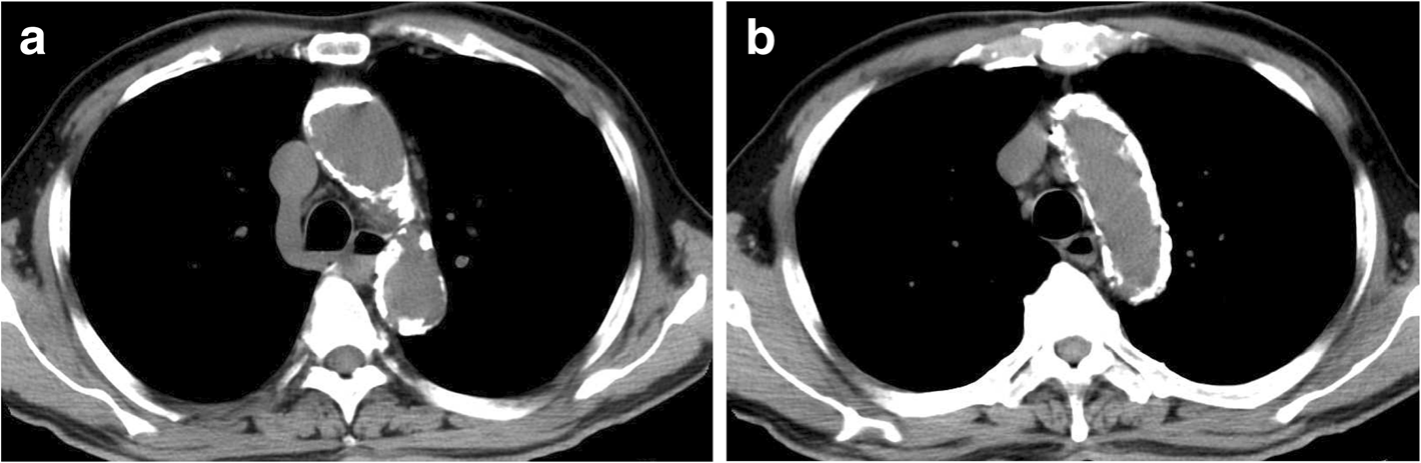
Computed tomography showing severe calcification of the ascending aorta (**a**) and aortic arch (**b**)

**Fig. 2:**
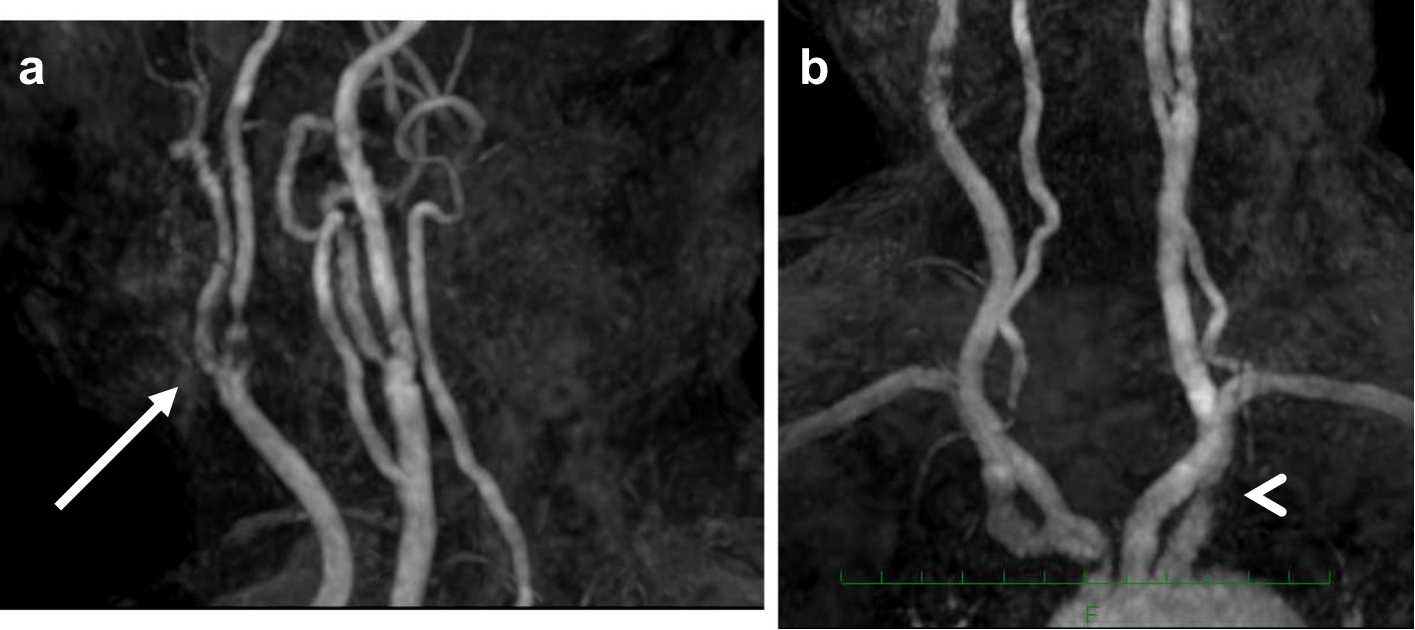
Magnetic resonance imaging showing stenosis of the right internal carotid artery (arrow) (**a**) and the left subclavian artery (arrowhead) (**b**)

A 79-year-old male was referred to our hospital with chest pain on exertion. Echocardiography demonstrated severe calcification of the aortic valve and severe aortic stenosis (aortic valve area, 0.7 cm^2^; maximum velocity, 4.8 m/s; mean pressure gradient, 50.4 mmHg). Coronary angiography showed no significant stenosis in the coronary arteries. Computed tomography revealed severe diffuse calcification from the ascending to the abdominal aorta. In particular, the aortic arch was shown to be circumferentially calcified and its side branches also had severe calcification (Fig. 1). Magnetic resonance imaging revealed more than 70 % stenosis in the brachiocephalic, right internal carotid, and left subclavian arteries (Fig. 2).

Because the aortic and iliac arteries were severely calcified, we chose not to perform transcatheter aortic valve replacement. Instead, conventional surgical aortic valve replacement was planned with bilateral axillary artery cannulation and short-term moderate hypothermic circulatory arrest for cross-clamping of the severely calcified ascending aorta. The operation was performed in the elective setting. Before sternotomy, 8-mm prosthetic grafts were anastomosed to the bilateral axillary artery for arterial cannulation. After sternotomy, a cardiopulmonary bypass was initiated with a venous return from the right atrium and a left ventricular venting from the right upper pulmonary vein. After the patient’s body temperature was decreased to 30 °C, systemic perfusion was temporarily arrested with the patient in the Trendelenburg position. The proximal ascending aorta with a less calcified site was opened in an oblique fashion. The inside aortic wall was inspected and a suitable site for cross-clamping was identified under direct vision [2]. The aorta was clamped with blood flushed from the arterial cannulas with a flow of 500 ml/min. The aortic valve was shown to be severely calcified, and was resected using a Cavitron Ultrasonic Surgical Aspirator (CUSA). A 21-mm bioprosthetic valve was replaced. An intimal calcified plaque near the clamped site was resected using a CUSA, then the aorta was closed with 4–0 polypropylene. The total cardiopulmonary bypass time was 63 min. The postoperative course was uneventful, and no neurological deficit occurred.

## Discussion

A severely calcified ascending aorta and arch are considered to increase the risk of a cerebral emboli occurring in patients undergoing aortic valve replacement. Several technical options have been used to avoid this complication, such as deep hypothermic circulatory arrest with or without ascending aortic replacement, endarterectomy of the ascending aorta, aortic inspection and cross-clamping during hypothermic circulatory arrest, and multiple arterial cannulation using the EndoClamp aortic balloon catheter [2–5]. In the present case undergoing aortic valve replacement, we selected bilateral axillary artery cannulation and short-term moderate hypothermic circulatory arrest for cross-clamping of the porcelain aorta to prevent neurological complications. We did not perform aortic replacement because the aortic arch and descending aorta were also severely calcified.

De Paulis et al. previously reported the usefulness of double-arterial cannulation for aortic valve replacement in three patients with porcelain aorta [5]. They adopted right axillary artery and femoral artery cannulation for double arterial cannulation, then endoclamped the aorta by a Foley occluding balloon inserted with a purse string suture into the distal part of the arch. After mild hypothermia was reached, unilateral antegrade cerebral perfusion was obtained. Although we agree with their concept for avoiding aortic clamping and protecting the brain against emboli from the proximal aorta, the present patient had multiple stenosis in the carotid and subclavian arteries so we used the bilateral axillary artery to secure the cerebral circulation.

Takami et al. reported the usefulness of aortic cross-clamping during short-term moderate hypothermic circulatory arrest in patients with a severely diseased ascending aorta [2]. They adopted the axillary or femoral artery for arterial cannulation, if the aorta had no space for cannulation. When patients were cooled to 30 °C, the aorta was opened with circulatory arrest. After inspection from the inside of the ascending aorta was complete, the aorta was carefully clamped at the debridement or endarterectomized site. We did not perform endarterectomy because a site with less atheromatous was identified in the intima of the ascending aorta under direct vision. In the present patient, the intraoperatively inspected inner aspect of the ascending aorta during circulatory arrest correlated well to the CT-scan. We believe that an aortic clamp with low-flow flush to an open space is helpful in diminishing injury to the intima and embolic debris traveling to the brain.

Bilateral axillary artery cannulation was useful not only for brain protection but also for a sufficient flow rate. We believe the combination of these is useful in patients with multiple stenosis in the arteries.

## Conclusion

In conclusion, we showed that bilateral axillary artery cannulation and short-term moderate hypothermic circulatory arrest for cross-clamping of the porcelain aorta is a suitable option to prevent neurological complications in patients with a severely calcified aorta and stenosis of its side branches who need aortic valve replacement.
